# Site-specific and time-course changes of postmenopausal osteoporosis in rat mandible: comparative study with femur

**DOI:** 10.1038/s41598-019-50554-w

**Published:** 2019-10-02

**Authors:** Chena Lee, Jeong-Hee Lee, Sang-Sun Han, Young Hyun Kim, Yoon-Joo Choi, Kug Jin Jeon, Hoi In Jung

**Affiliations:** 1 Department of Oral and Maxillofacial Radiology, Yonsei University College of Dentistry, Seoul, Republic of Korea; 2 Department of Preventive Dentistry & Public Oral Health, Yonsei University College of Dentistry, Seoul, Republic of Korea

**Keywords:** Osteoporosis, Outcomes research

## Abstract

Although the effects of osteoporosis on the skeleton are well studied, site-specific and long-term studies on the mandible are still lacking. This study investigated the time-course changes of the bone microarchitecture in the mandibular condyle in comparison to the corresponding changes in the alveolar bone, body of the mandible, and femur. Thirty-six 11-week-old female Sprague-Dawley rats were divided into ovariectomized (OVX) (24 rats) and sham (12 rats) groups. The right femur and mandible were obtained from 6 OVX rats and 3 sham rats at 8, 12, 26, and 36 weeks after surgery, respectively. The histomorphometric analysis was performed using micro–computed tomography and histologic assessments from the (1) distal femur; (2) the alveolar bone and (3) the body of the mandible; (4) the subchondral and (5) the central region of the condyle. The Brown-Forsythe test was used to verify the assumptions for statistical analysis, and the Mann-Whitney U test was then performed. The mandibular condyle showed increased trabecular bone in both the OVX and sham groups, while the bone density was reduced in the distal femur and the mandible interradicular septum and body. When comparing the OVX group to the sham group, only the central condyle showed a significant reduction in bone density at 36 weeks. Osteoporosis behaves in different manners in different parts of the skeleton, and clinicians should be aware that patients displaying osteoporotic changes in the mandible are expected to show severely advanced bone mineral density reduction in other bones, such as the femur.

## Introduction

Osteoporosis is a skeletal disorder caused by disruption of the mineral density and microstructure of the bone^1^. A recent study reported that although bone mineral density (BMD) has been found to decrease in both the femur and the lumbar spine in patients with osteoporosis, the amount of the decrease in each region did not show a significant correlation^2^. Previous research has concluded that it is difficult to estimate the risk of hip fracture due to osteoporosis based on other body parts, such as the spine or forearm^3^.

Mandibular bone affected by osteoporosis is also known to behave differently from other body parts. However, site-specific evaluations within the mandible and long-term investigations of changes in the mandible have rarely been performed. The mandible is a very complex structure, from both developmental and functional perspectives, compared to other skeletal structures. It is composed of joint, tooth-bearing, and muscle-attached areas, and each region shows a distinct trabecular bone pattern due to differences in function and mechanical stress^4^. In this context, each part of the mandible is expected to show completely different bone microarchitectural changes in response to osteoporosis. In particular, the mandibular condyle is a morphologically and functionally unique joint structure^5^. Despite its distinctive features, the impact of osteoporosis on the mandibular condyle has scarcely been studied.

For assessing osteoporosis in human beings, many researchers have attempted to predict systemic osteoporosis based on the mandibular cortex width shown in dental panoramic radiographs^6,7^, because panoramic radiography is a commonly used modality and regular examinations are performed periodically on patients with little cost. However, panoramic radiography has limitations in assessing osteoporosis of the jaw, since the image presents the mandible and maxilla superimposed with various head and neck anatomic structures, such as the cervical spine or ghost shadow of the opposite side of the mandibular angle^6^. In addition, studies on osteoporosis in the jaw have been based on the premise that osteoporosis in the mandible corresponds to systemic osteoporosis elsewhere in the body. It is difficult to investigate the effect of systemic osteoporosis on the jaw in human beings. In addition, there is no secure evidence for a link between mandible BMD and estrogen deficiency–induced osteoporosis, as Nicolielo and colleagues stated in their review paper^5^. The degree to which BMD decreases due to osteoporosis in the mandible and how it is related to other skeletal bones should be investigated first, before finding a method of predicting systemic osteoporosis from mandibular bone changes on panoramic radiography.

Furthermore, the study results that have been reported do not show good agreement. Hsu *et al*.^8^ reported that the microarchitecture of trabecular bone in mandible showed alterations at 12 weeks after ovariectomy, while Tanaka *et al*.^9^ reported that the alveolar bone of rats started to show changes a year after surgery. A recent review article also stated that limited evidence still exists regarding condyle resorption in response to estrogen deficiency–induced osteoporosis, with a paucity of controlled studies^5^.

Therefore, it is important from a clinical perspective to clearly characterize the associations of individual mandibular regions with systemic osteoporosis. For studying estrogen deficiency–induced osteoporosis, ovariectomized (OVX) rats are a generally accepted model, approved by the Food and Drug Administration^10^. Previous studies have confirmed that trabecular bone volume of the tibia, femur, and lumbar spine decreased within 12 weeks after surgery in this animal model^9–12^.

This study was conducted to investigate the timing of changes in the microarchitecture of the mandibular condyle in rats due to osteoporosis, with periodic post-ovariectomy observations. Furthermore, bone changes in multiple other regions of the mandible, including the mandibular alveolar bone and body, were compared to the corresponding changes in the distal femur.

## Materials and Methods

### Animal care

Thirty-six 11-week-old female Sprague-Dawley rats (approximate weight, 230–260 g), were obtained from a breeder (Orientbio Inc., Seongnam, Korea). After 7 days of acclimatization, 12-week-old female rats were housed in a temperature of 25 °C with a 12-hour light and 12-hour darkness cycle and allowed free access to water and a standard rodent diet. The experiment was conducted in the Laboratory Animal Unit, Yonsei Biomedical Research Institute, Yonsei University College of Medicine. Prior to the experiments, the animal experiment protocol was approved (approval number 2016–0325) by the Institutional Animal Care and Use Committee of Yonsei University Health System, and all experiments were performed in accordance with the University’s Guidelines for Animal Experimentation and ARRIVE guidelines.

### Surgical procedure

The 36 rats were divided randomly into 2 groups: 24 rats underwent a bilateral ovariectomy (the OVX group) and the remaining 12 rats underwent sham surgery (the sham group) to simulate surgical stress based on a standardized procedure from a previous publication^13^. The procedure was performed by qualified veterinary surgeons. In brief, inhalation anesthesia was induced via 3–4% isoflurane and 1–2 L/min of oxygen in an induction chamber. Then, the rat’s hair was shaved from the back to both flanks, from the femur to rib cage. A 2-cm ventral incision was made, the fat pad was moved, and the ovaries were excised around the perinephric region. After removing the ovaries, the muscle layer was closed with a single-stitch suture (Vicryl 5/0, Ethicon Inc., Somerville, MA, USA) and the skin layer was closed with black silk (5/0, Ailee co., LTD., Busan, Korea). The control group underwent a similar standard procedure, except the ovaries were kept intact. Meloxicam (2 mg/kg; Metacam, Boehringer Ingelheim, Germany) was given to rats postoperatively for 3 days for pain relief. Likewise, enrofloxacin (2.5 mg/kg; Baytril 50 Inj., Bayer, Germany) was given to prevent infection.

### Sample preparation

Six rats in the OVX group and 3 rats in the sham group were euthanized at 8, 12, 26, and 36 weeks post-surgery, respectively. Euthanasia was done by carbon dioxide gas with 3% isoflurane with pure oxygen as the carrier for isoflurane anesthesia. The mandible and femur were harvested and the remaining soft tissue was carefully removed. The mandibles were split in half and all excised samples were fixed in 10% neutral buffered formalin solution for 2 days.

### Histomorphometric analysis using micro–computed tomography

The right side of the split mandible and the right side of the femur samples were scanned with a micro–computed tomography (CT) system (SkyScan 1173; Bruker-CT, Kontich, Belgium) using the following settings: total rotation, 180°; step size, 0.3°; source settings, 130 kV and 60 μA; and exposure time, 0.5 s per step. Raw images were acquired with pixel dimensions of 15 μm × 15 μm, and were used to reconstruct three-dimensional images in the Skyscan software package (NRECON, version 1.6.10.4, SkyScan, Kontich, Belgium), which were analyzed using CTAn version 1.12.0 (SkyScan, Kontich, Belgium).

**Figure 1:**
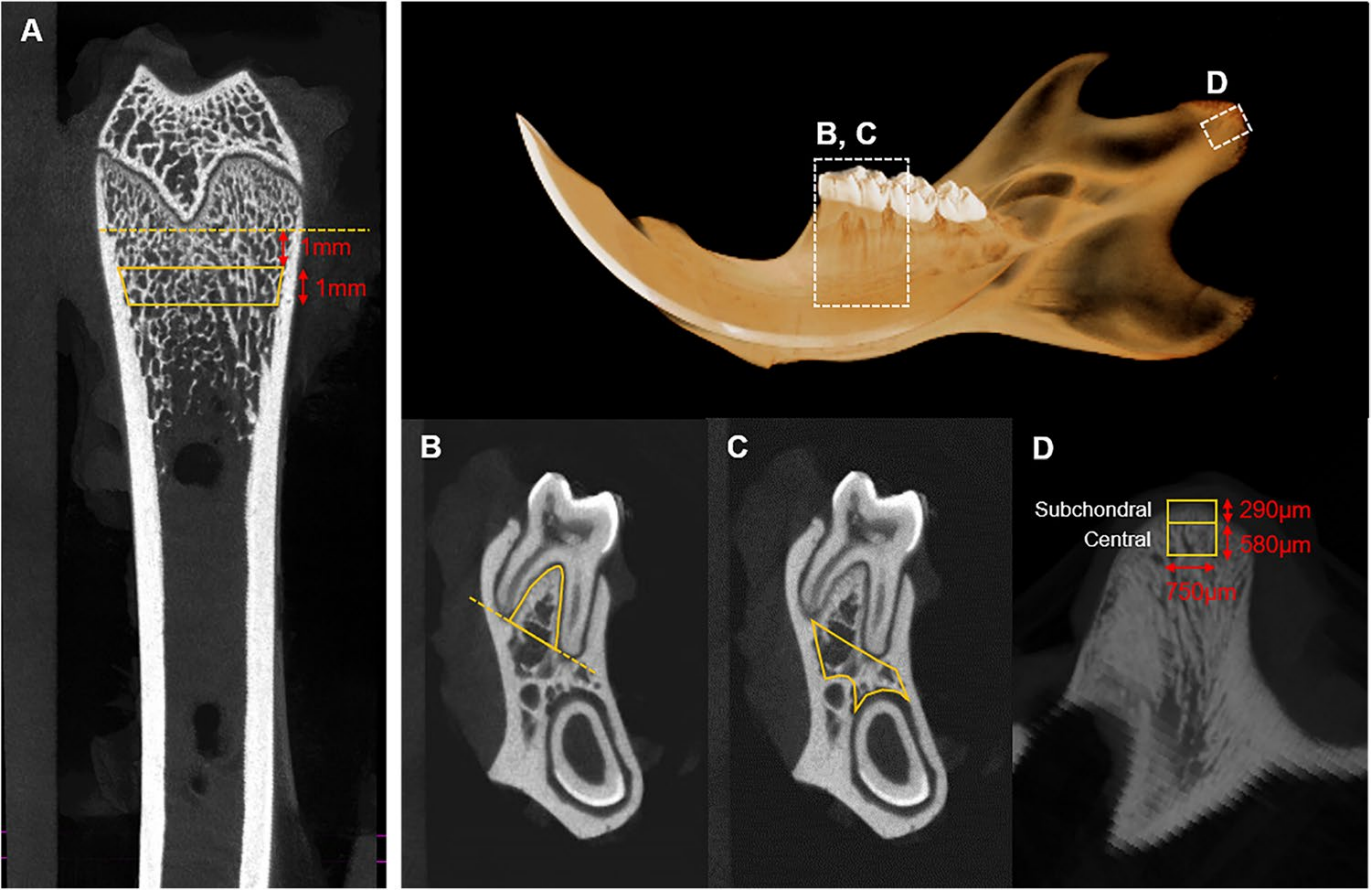
Region of interest (ROI) selection in micro–computed tomography and histology assessments. (**A**) Trabecular region of the distal femur (the yellow outline represents the ROI on the femur). (**B**) The interradicular septum between the medial and distal roots of the first molar. (**C**) The mandibular body region below the molars from the first molar and second molar. (**D**) The subchondral (center of condyle surface to the depth of 290 µm) and central regions (depth of 290–580 µm) of the mandibular condyle.

All regions of interest (ROIs) were limited to trabecular regions; cortical bone was discarded due to its particular sensitivity and lack of comparability to osteoporosis in humans^14^. ROIs on the right side of the distal femur were selected, and a longitudinal extension of 1 mm was performed to avoid involving the primary spongiosa and new bone growth^15^ (Fig. 1A). ROIs of the mandible were selected in 4 regions that were modified from previous reports^16–18^. The first of the 4 regions in the mandible was manually defined in the trabecular bone area of the interradicular septum of the first molar (M1) (Fig. 1B). The border of the ROI was established by a straight line between the medial and distal root apexes of M1. The second ROI was acquired from the mandibular body positioned below the molars from M1 and the second molar (Fig. 1C). The mandibular condyle was divided into 2 ROIs: the region connected to cartilage was referred as the subchondral region, while the region beneath the subchondral region was referred to as the central region (Fig. 1D).

The following parameters of bone density and trabecular structure were carefully evaluated: (1) BMD; (2) bone volume/tissue volume (BV/TV; %), which refers to the ratio of the segmented bone volume to the total volume of the ROI; (3) trabecular thickness (Tb.Th; mm), which is a measure of the mean thickness; (4) trabecular separation (Tb.Sp; mm), which is a measure of the average distance of the marrow cavities between the trabeculae; and (5) trabecular number (Tb.N; 1/mm), which indicates the average number of trabeculae per unit length.

### Histological assessment

After obtaining micro-CT scans, the samples were fixed in standard formalin solution with 10% neutral buffer for 2 weeks. The fixed sample then underwent a decalcification process using Calci-Clear Rapid (National Diagnostics, Atlanta, GA, USA). Furthermore, dehydration using a graded ethanol series of 70%, 95%, and 100% was performed, and the samples were soaked in xylene and then embedded in paraffin. The mandibular body and molar region were sectioned in the mesial-distal direction, and the distal femur and the condyle were sectioned in the longitudinal direction in series of 3-µm thickness using a microtome (Reichert-Jung, Heidelberg, Germany). The samples were stained with Masson trichrome to discriminate bone from soft tissue. The series of sections were observed using a light microscope (Olympus BX51; Olympus Co., Ltd., Tokyo, Japan) and examined using a slide scanner (3D-Histech, Budapest, Hungary).

### Statistical analysis

All parameters are reported as the mean ± standard deviation (SD) of each group. The homogeneity of variance between the control and experimental group was confirmed using the Brown-Forsythe test. The Mann–Whitney U test was performed to compare the distribution of the trabecular bone microarchitectural parameters of the mandibles and distal femur between the OVX and sham groups. A P-value of less than 0.05 was considered to indicate statistical significance. Data were analyzed using SPSS version 23.0 (IBM Corp., Armonk, NY, USA). We determined the sample size based on the effect size of BMD measurements in previous research with a power (1- β error) of 0.8 and an alpha error of 0.05 using G*Power 3.1.3 statistical software^19,20^.

## Result

**Figure 2:**
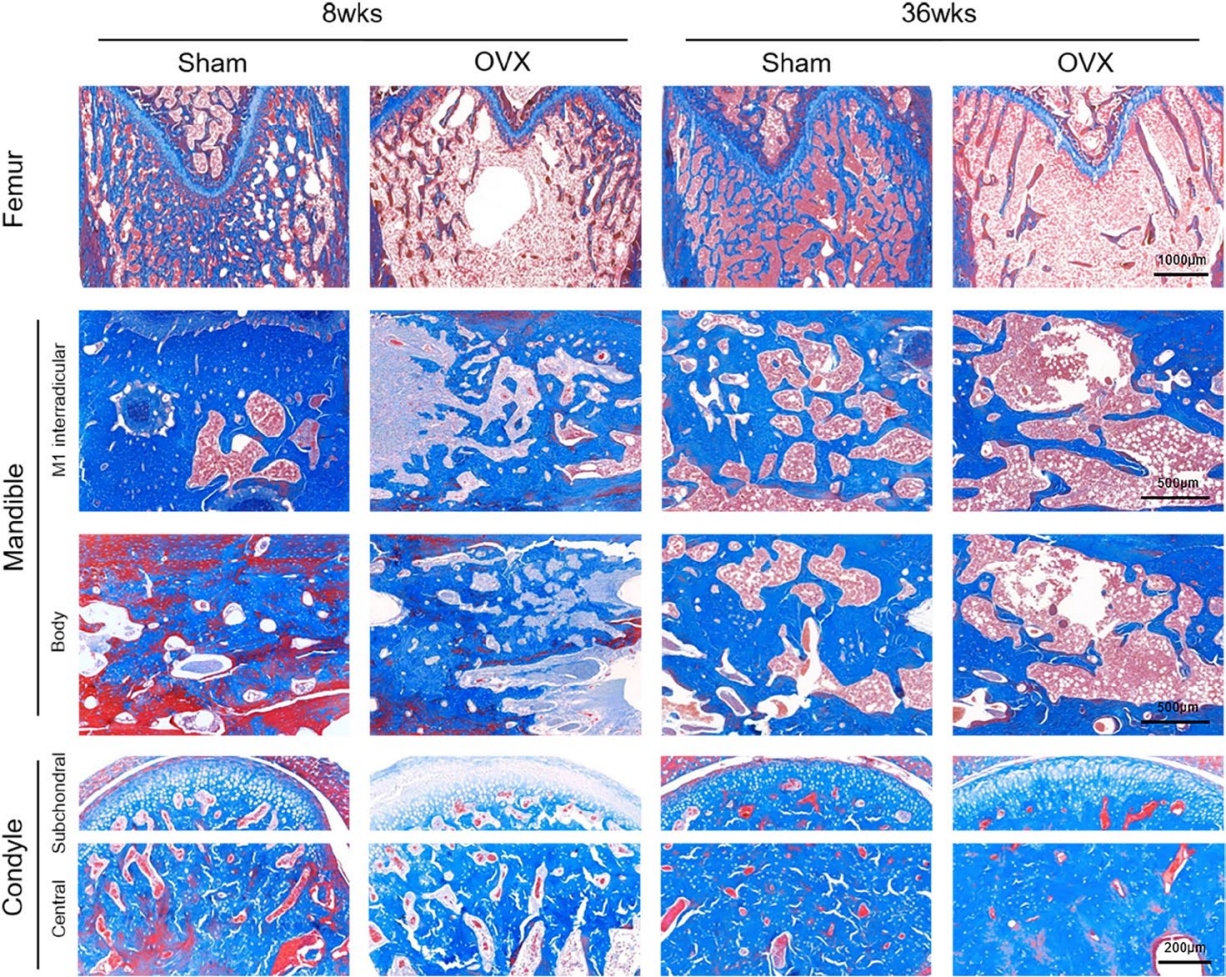
Photomicrographs of Masson trichrome–stained sections in rat femur, mandible, and condyle at 8 and 36 weeks after surgery. The blue-stained area indicates bone and red indicates soft tissue. In femur, the trabecular bone pattern is significantly lower in ovariectomized group than in the sham surgery group. The condyle shows a significant bone increase in both groups at 36 weeks post-surgery.

**Figure 3:**
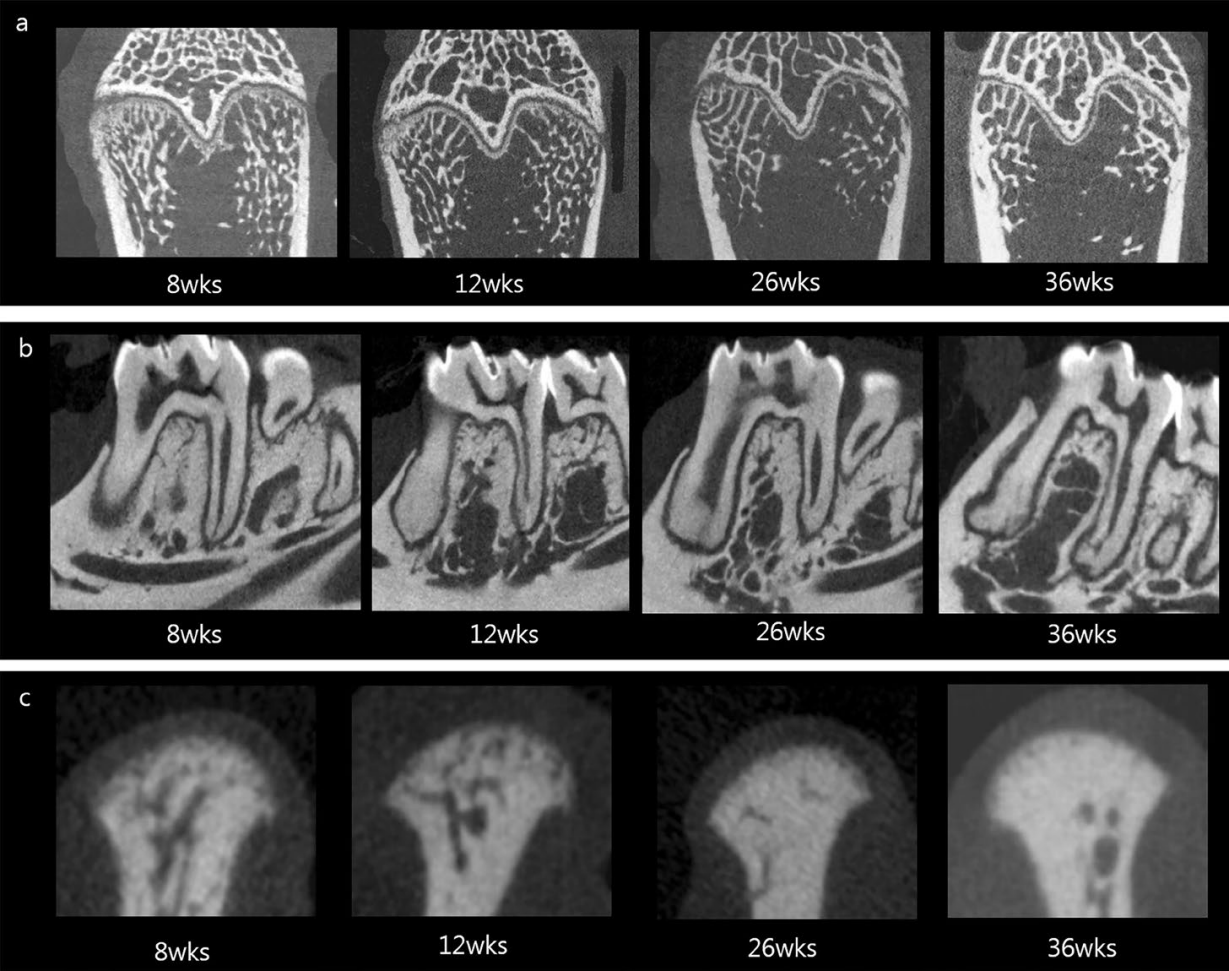
Micro–computed tomography images of the ovariectomized group over time. (**a**) The femur shows significant osteolytic changes from the center area and the changes progressed over time. (**b**) The mandible shows a slight bone decrease in the interradicular area at 36 weeks. (**c**) Both the subchondral and central condyle region present a gradual increase of bone density.

Microscopy and micro-CT images revealed that the mandibular condyle showed increased trabecular bone in both the OVX and sham groups, unlike the femur and mandible areas (Figs 2 and 3). On the contrary, in the femur, the OVX group showed a significant decrease in trabecular bone compared to the sham group at week 36. Trabecular bone was notably reduced in the distal femur and minimally reduced in the mandible at both the M1 interradicular and body regions.

**Figure 4:**
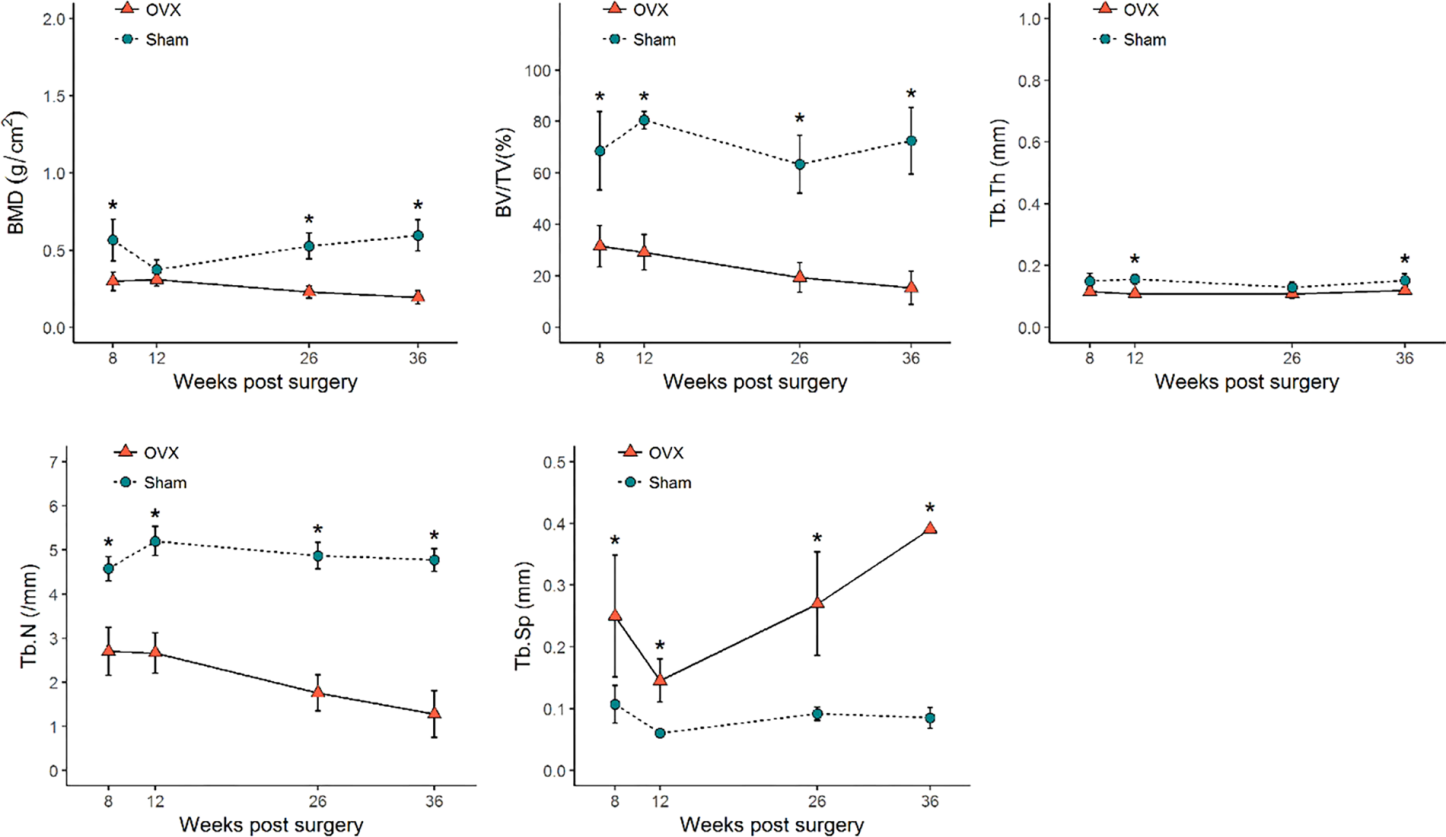
Bone microarchitectural parameters in the distal femur. Values are mean ± standard deviation. BMD, bone mineral density; BV/TV, bone volume fraction; Tb.Th, trabecular thickness; Tb.N, trabecular number; Tb.Sp, trabecular separation. * denotes a significant difference between the OVX group and the corresponding values of the sham group (Mann–Whitney U test, P < 0.05). OVX, ovariectomized.

**Figure 5:**
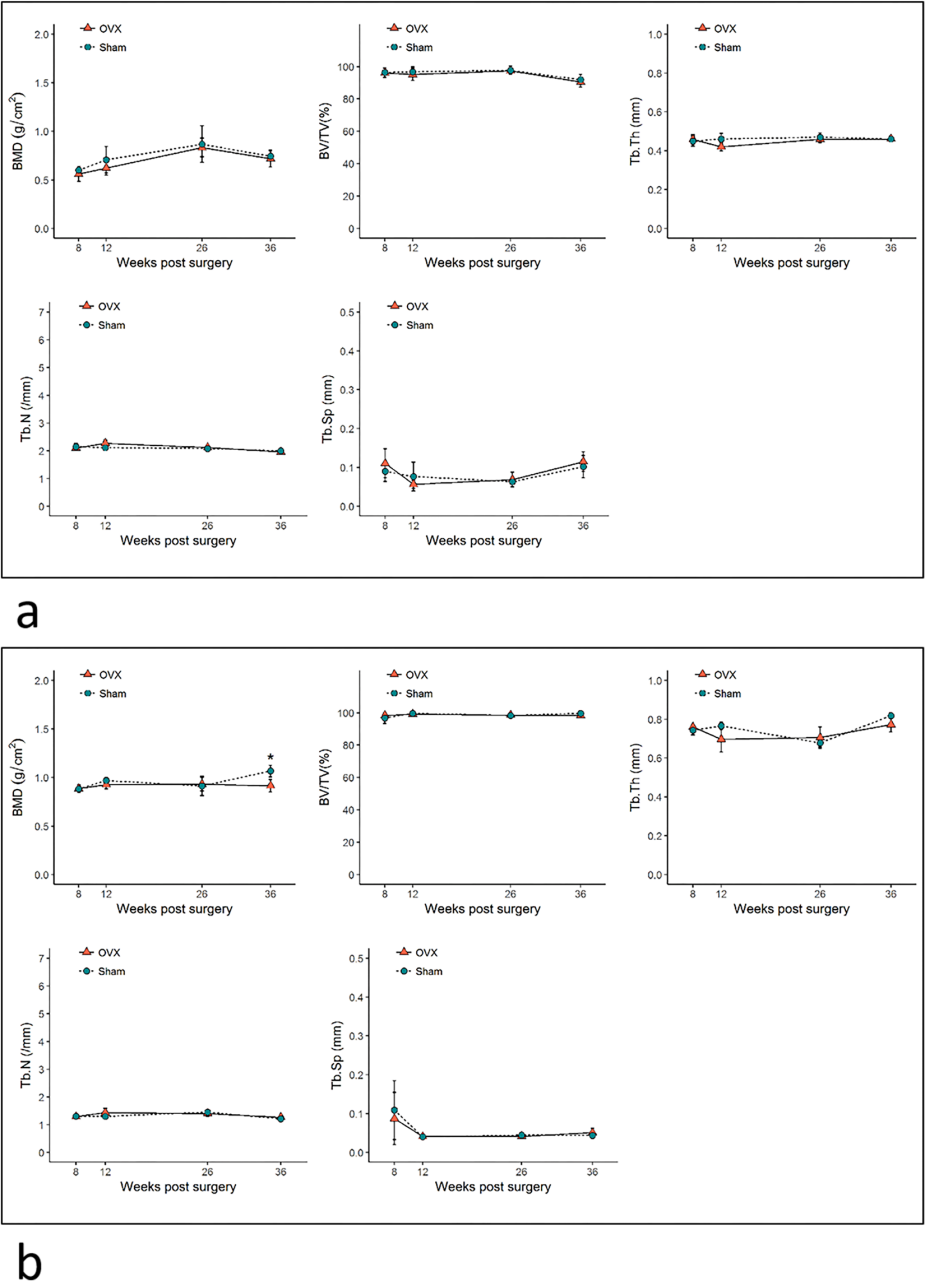
Bone microarchitectural parameters in the (**a**) subchondral region of the condyle and (**b**) central region of the condyle. Values are mean ± standard error. BMD, bone mineral density; BV/TV, bone volume fraction; Tb.Th, trabecular thickness; Tb.N, trabecular number; Tb.Sp, trabecular separation. * denotes a significant difference between the OVX group and the corresponding values of the sham group (Mann–Whitney U test, P < 0.05). OVX, ovariectomized.

**Figure 6:**
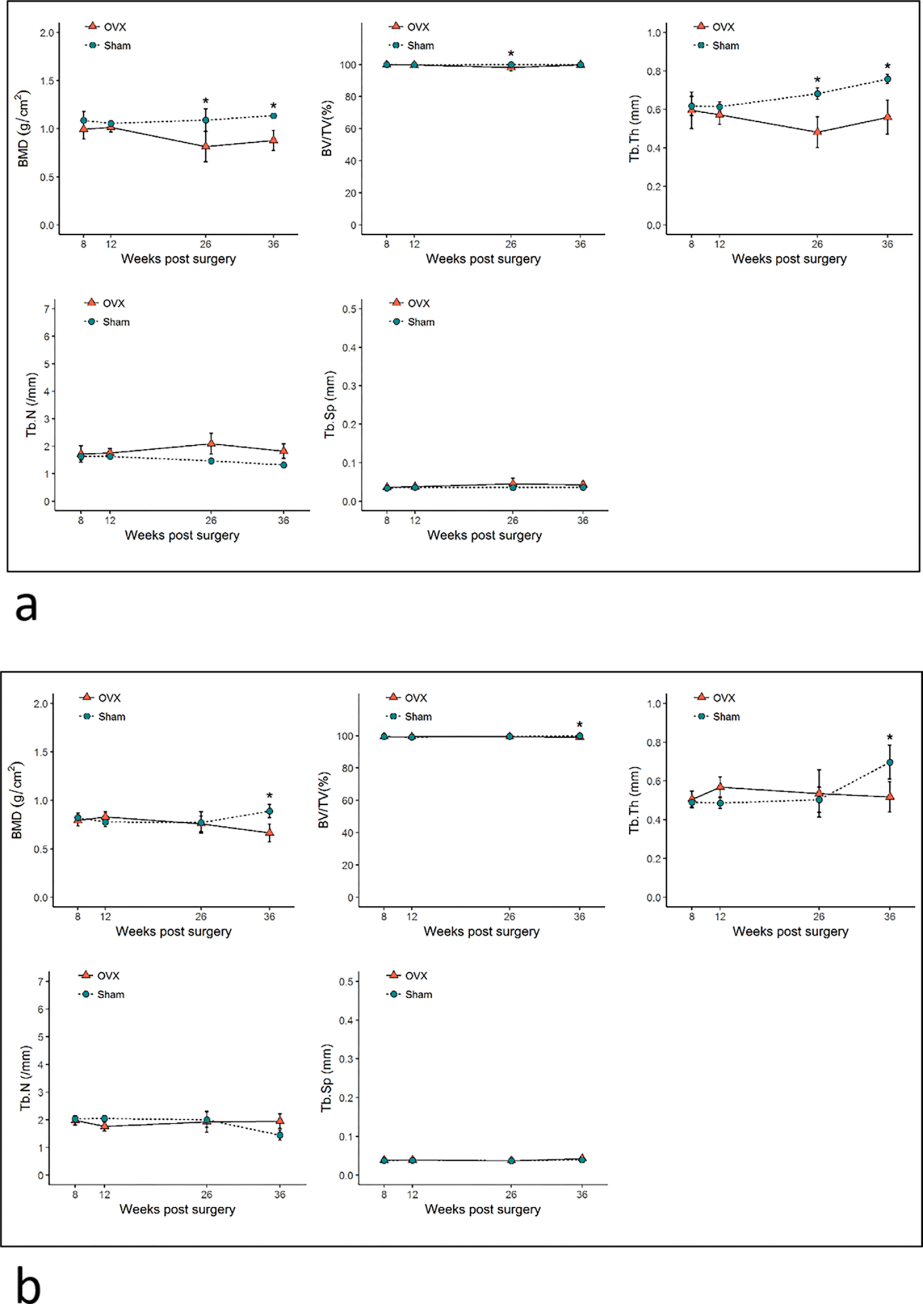
Bone microarchitectural parameters in the (**a**) interradicular septum of the first molar and **(b**) the mandibular body. Values are mean ± standard error. BMD, bone mineral density; BV/TV, bone volume fraction; Tb.Th, trabecular thickness; Tb.N, trabecular number; Tb.Sp, trabecular separation. * denotes a significant difference between the OVX group and the corresponding values of the sham group (Mann–Whitney U test, P < 0.05). OVX, ovariectomized.

The quantification of bone microarchitectural changes (mean ± SD) in the OVX and sham groups is shown in Figs 4–6. In the distal femur, significant bone microarchitectural changes were detected between the OVX and sham groups starting at 8 weeks after ovariectomy. At week 36, the last observation point, the BMD value in the OVX group was 67.8% of that in the sham group (Fig. 4).

All regional sites of the mandible showed delayed or absent BMD reduction compared to the distal femur. The mandibular condyle did not show noticeable changes in trabecular bone in either the subchondral or central region. Only the central region of the condyle at week 36 showed a significant difference in BMD, with a 14.0% difference between the OVX and sham groups (Fig. 5).

In the M1 interradicular region of the OVX group, significant bone changes were observed starting at week 26 post-surgery. By week 36, the BMD of the M1 interradicular region in the OVX group was reduced by 23.0% and Tb.Th was reduced by 26.7% compared to the sham group. The mandibular body also showed reductions in BMD, BV/TV, and Tb.Th (25.0%, 0.7% and 26.0%, respectively) compared with the sham group. Tb.N and Tb.Sp did not show significant differences between the OVX and sham groups in either the mandibular body or the M1 interradicular area (Fig. 6).

## Discussion

The impact of postmenopausal osteoporosis has long been studied, especially its effects on weight-bearing skeletal structures, such as the femur and spine in ovariectomized animals^9,10^. However, bone microarchitectural changes in the jaw from estrogen deficiency have not been studied enough to reach a consensus, and studies of ovariectomized rats have presented conflicting results^8,16–18^.

Thoroughly evaluating changes in mandibular bone microarchitecture is challenging due to the complex anatomy of the mandible. In fact, due to the narrow and irregular anatomy of the mandible, different ROIs tend to be used in different experiments. Some studies included the body area within the interradicular region, which was supposed to be separately analyzed^20^. In addition, osteoporosis-induced changes in the mandible have been reported to appear later than those in other regions of the skeleton^8,16^, making it difficult to determine the appropriate time point for analysis. Most studies performed single-time-point analyses due to cost and time constraints^19–22^.

In order to further elucidate these controversial issues, the current study analyzed periodic changes in bone microarchitecture in the mandible at 8, 12, 26, and 36 weeks after ovariectomy. Furthermore, different functional regions in the mandible, including a tooth-bearing region (M1 interradicular region), a muscle-attaching basal bone area (the mandibular body), and a joint component (subchondral and central condyle), were precisely established as individual ROIs. These results were compared with those of the femur, which has been relatively thoroughly investigated in many previous studies. Thus, the findings of this study are noteworthy in that we performed an analysis at several time points over a relatively long period at multiple sites of the mandible.

This study confirmed that the mandible, especially the interradicular and body areas, were less sensitive to systemic osteoporosis than the femur, which is consistent with several previous studies^21,23^. The bone mass reduction in the interradicular region was somewhat more prominent than in the body area. Johnston *et al*.^24^ also reported that the first molar interradicular septum is known to be the most sensitive site to osteoporosis due to estrogen deficiency.

The bony area surrounding the molars in the mandible has been commonly used as an ROI in previous studies due to its importance during dental implant surgery. An implant fixture may not be loaded until successful osseointegration is confirmed, and the duration of osseointegration is closely related to the bone quality^25^. More specifically, bone microarchitecture, such as trabecular thickness, number, and distribution, affects the stability and long-term success of dental implants^26^. As Johnston and Ward stated, the deterioration of the alveolar bone in OVX rats resembles osteoporotic changes in postmenopausal women^24^. It should be kept in mind that animal studies cannot directly reflect biological changes of the human body, but the present study implies that patients diagnosed with osteoporosis through a femoral examination may not display as poor bone quality as expected in the mandible. In addition, the tooth-bearing area was the first to show osteoporotic changes, and showed the greatest extent of osteoporotic changes, in comparison to the mandibular body and condyle areas.

It is interesting that the mandibular condyle showed a completely different response to estrogen deficiency from the alveolar bone and mandibular body. The subchondral region was completely protected from osteoporotic changes until week 36 post-ovariectomy. In contrast, Tanaka *et al*.^17^ reported that the subchondral area was under the transient influence of estrogen deficiency. This discrepancy might be due to the use of different time points in the analyses. The last time point after sacrifice was much earlier in their study (8 weeks postoperatively) while the current study traced OVX rats for a longer period. Since bone changes after 36 weeks post-ovariectomy have not been studied, it may be useful to perform further examinations with a longer follow-up period.

In the central condyle region, BMD was the only factor that showed a statistically significant difference between the OVX and sham group at week 36. Other microarchitectural parameters did not show significant differences between the OVX and sham groups. Interestingly, the difference occurred due to an increase in BMD in the sham group, rather than a decrease in BMD in the OVX group. Tanaka *et al*. suggested that ovariectomy suppressed bone gain, rather than accelerated bone resorption, in the central condyle area^17^. This study also showed that the BMD of the central condyle in the OVX group was slightly decreased at the last follow-up period, 36 weeks, while the sham group showed increase in the value. Jiao *et al*. reported that the trabecular bone density of the condyle in healthy female rats continuously increased even after the growth spurt^27^. These previous reports are somewhat consistent with the present study in that the trabecular bone pattern became noticeably denser on microscopy and micro-CT in the condyle area.

The progression of condylar resorption stimulated by estrogen deficiency has not been clearly characterized, and has been assumed to reflect a dysfunctional remodeling process according to a systematic review of the previous literature in humans^5^, although the authors acknowledged that there was insufficient evidence to support this hypothesis. Few studies using a rat model have focused on condylar bone changes induced by estrogen deficiency and even those studies reported inconsistent results^17,27^. It is suspected that the follow-up period for those previous experiments was not sufficient for the condyle to present a certain degree of bone change, as was demonstrated in the current study. We found that 36 weeks post-ovariectomy was the initial point of a significant change in trabecular microarchitecture in the condyles of rats with estrogen deficiency–induced osteoporosis, compared with the normal group. It is necessary to conduct microarchitecture analyses at more advanced time points to confirm the time course of mandibular condyle resorption.

## Conclusions

In conclusion, osteoporosis behaves in different manners in different parts of the skeleton. The mandible presents a substantially delayed and less intense response in terms of BMD loss compared to the femur in a rat model of osteoporosis. The subchondral region of the condyle was the least influenced by osteoporosis in the mandible, while the first molar interradicular septum was a more sensitive area in terms of showing microarchitectural changes. Clinicians should be aware that patients displaying osteoporotic changes in the mandible are expected to show severely advanced BMD reduction in other bones, such as the femur.
